# Designing and developing a mobile app (BeBo) in a randomized controlled trial study to promote breastfeeding among Vietnamese mothers

**DOI:** 10.1186/s13006-023-00543-7

**Published:** 2023-01-19

**Authors:** Thi Thuy Duong Doan, Trung Chuyen Tran, Ngoc Minh Pham, Yun Zhao, Thi Phuong Hoa Dinh, Nguyen Xuan Hoai, Andy Lee, Colin Binns, Thi Thu Ha Bui

**Affiliations:** 1 Faculty of Social Sciences, Behaviour and Health Education, Hanoi University of Public Health, 1A Duc Thang Street, Bac Tu Liem District, Hanoi, 10000 Vietnam; 2 School of Public Health, Curtin University, Bentley, Western Australia 6102 Australia; 3 Faculty of Information Technology, Department of Hanoi University of Mining and Geology, 18 Vien Street - Bac Tu Liem District, Hanoi, 10000 Vietnam; 4 Thai Nguyen University of Medicine and Pharmacy, Thai Nguyen City, 250000 Vietnam; 5 Faculty of Information Technology, HUTECH University, Ho Chi Minh City, 700000 Vietnam

**Keywords:** mHealth, Smartphone, App, Breastfeeding, Vietnam

## Abstract

**Background:**

Breastfeeding should begin as soon as possible after birth and continue exclusively to 6 months of age. In Vietnam, as in many other countries, breastfeeding is decreasing because of modern lifestyles and the promotion of infant formula. It is important to provide mothers, family members, and the community with the knowledge and strategies to improve breastfeeding rates. Smartphones are almost ubiquitous in Vietnam and of the potential to provide information about breastfeeding. This study aimed to document the process of designing and developing a mobile app to increase breastfeeding rates in Vietnamese women.

**Methods:**

We used a four-step mixed methods approach with a literature review, formative research (22 in-depth interviews and 49 self-administered online questionnaires), and testing of prototype apps (3 focus groups discussion and external experts). Formative research and focus group discussion involved 99 participants. Finally, the revisions of the app were tested. All of the formative research was undertaken in Hanoi in 2019–2020. Target behaviors followed by key determinants, to improve breastfeeding self-efficacy were studied and this information was then applied in developing the messages and library content. Barriers and facilitators to breastfeeding were identified from literature reviews and qualitative research. The messages were targeted at not only mothers but also included fathers, mothers-in-law, or families.

**Results:**

Mothers were mostly concerned about the initiation of breastfeeding, preventing and reducing difficulties encountered during breastfeeding, and nutrition for breastfeeding mothers. Mental health and well-being in the postnatal period are also concerns. Three key features to be included in the app were identified from the formative research: (1) notifications; (2) an information library; and (3) a searching function. The research found that the app should be installed during pregnancy rather than after delivery (81% vs 17%, respectively). Notifications that convey breastfeeding messages should be sent 2–3 times per week.

**Conclusion:**

The development of the app followed a best practice approach, including the involvement of stakeholders and grounding in behavior change theory. The next step is to evaluate the effectiveness of the BeBo mobile app in a well-conducted randomized controlled trial.

**Trial registration:**

ACTRN12619000531112.

## Background

Vietnam is a middle-income country in Southeast Asia with a population estimated at 96.9 million inhabitants, with 35% living in urban areas [1]. About 95% of Vietnamese mothers have at least one antenatal visit and deliver with the support of a healthcare professional [2]. Despite 96.4% of Vietnamese women breastfeeding their child at any time, only 26.5% of infants had early initiation of breastfeeding within the first hour of birth and 24.3% were exclusively breastfed during the first 6 months of their lives, assessed by 24-hour recall [3]. While an increase in breastfeeding rates has been observed in other developing countries [4], it continues to decline in Vietnam, particularly in urban areas [5] and among mothers who had a cesarean section [5, 6].

Breastfeeding rates are influenced by many factors related to both the family and the community. Mothers are central to breastfeeding and their decisions have the most influence on initiating and maintaining breastfeeding [7]. A mother’s belief in her ability or self-confidence results in successful breastfeeding termed ‘self-efficacy’ [8, 9]. Breastfeeding self-efficacy is influenced through (a) performance accomplishments, including previous experience with breastfeeding, (b) vicarious experience, such as seeing other women breastfeeding successfully, (c) verbal persuasion, through encouragement and knowledge from influential others, and (d) physiologic responses to pregnancy and delivery, such as depression, anxiety, and fatigue. Those are modifiable factors that practitioners can target to improve breastfeeding rates [10, 11].

Recent successful interventions using mobile technologies have been shown to improve exclusive breastfeeding through appropriate mobile Health (mHealth) applications in high-income and other Asian countries [12, 13]. Most of the interventions used websites, e-mail, text messages, and/ or phone calls [12, 14, 15]. Recently, the mobile app has been used to improve interaction with mothers and personalized content [16]. However, most apps for infant feeding have been rated as poor quality [17], lacking evidence-based recommendations, and without a rigorous evaluation of effectiveness [18].

The popularity of smartphones in Vietnam provides an opportunity for implementing mHealth interventions, particularly in urban areas with 146 million mobile phone connections (150% of the population), and 68 million internet users (70% of the population) [1]. Half of the internet users are women (52%) [19]. We searched and found in the app store, some mobile apps related to pregnancy and child care in the Vietnamese language. However, there was only one app that provided information on breastfeeding, maternal health, and childcare. This app, however, also provided advertisements for infant formula companies. Information provided by the app was from unreliable resources and conflicted with the guidelines of the Ministry of Health and internationally accepted best practices in infant nutrition. There was no study on the effect of the app on breastfeeding rates.

In this paper, we describe the development of a mHealth app optimized to improve breastfeeding rates in urban areas in Vietnam. Based on our research and results, we suggest processes and recommendations for developing and evaluating mHealth apps adapted for use in low- and middle-income countries.

## Methods

### Study overview

We developed a prototype mHealth app, which we named BeBo, which is targeted toward Vietnamese mothers to increase breastfeeding rates. The name “BeBo” was selected as it mimicked infant babbling and baby jargon. We then designed a two-arm, parallel triple-blinded randomized controlled trial to evaluate the effectiveness of the “BeBo” app. Details of the study protocol have been published [20]. The study protocol was approved by the Curtin University Human Research Ethics Committee (Ref: HRE2019–0143-03) and the Ethical Review Board for Biomedical Research, Hanoi University of Public Health (Ref: 28/2019/YTCC-HD3). The trial’s registration number is ACTRN12619000531112.

Data were collected in Hanoi from May 2019 to March 2020.

### App development process

The BeBo team was composed of researchers and practitioners in health promotion, breastfeeding, infant nutrition, and maternal and child health care with experience in developing infant feeding guidelines from both Vietnam and Australia together with a software developer and a designer. The team had weekly meetings over the internet to review the development of the intervention.

The development process was adapted from the behavioral intervention technology model to ensure replicability [21]. This model was applied to designing both the conceptual and technological architecture of the app. It answered the questions of why, what, how, and when [21] during the development process as outlined below.

- Establishing the intervention aims (WHY): (a) to increase breastfeeding rates (b) be able to evaluate its effectiveness in improving breastfeeding rates through a randomized controlled trial.
- Selecting behavioral strategies to improve knowledge of mothers and aid shared decision (HOW): (a) to improve knowledge and enhance breastfeeding self-efficacy among mothers, (b) to promote shared information and involvement in decision-making on exclusive breastfeeding to family members and healthcare providers.
- Designing app elements to deliver selected behavioral strategies (WHAT): We identified the main messages delivered in form of push notifications. This element was the most important part of the success of the intervention [22, 23]. Recommended steps for developing and pretesting a text messaging program for health behavior change were adapted and integrated into the development of notifications [24]. Other app features were decided from the formative research as described below. The BeBo app prototype was delivered in the Vietnamese language.
- Defining when and under what conditions the app would be used (WHEN): time for installing the app, frequency and timing for generating push notifications, and expected information in the library content.

**Fig. 1:**
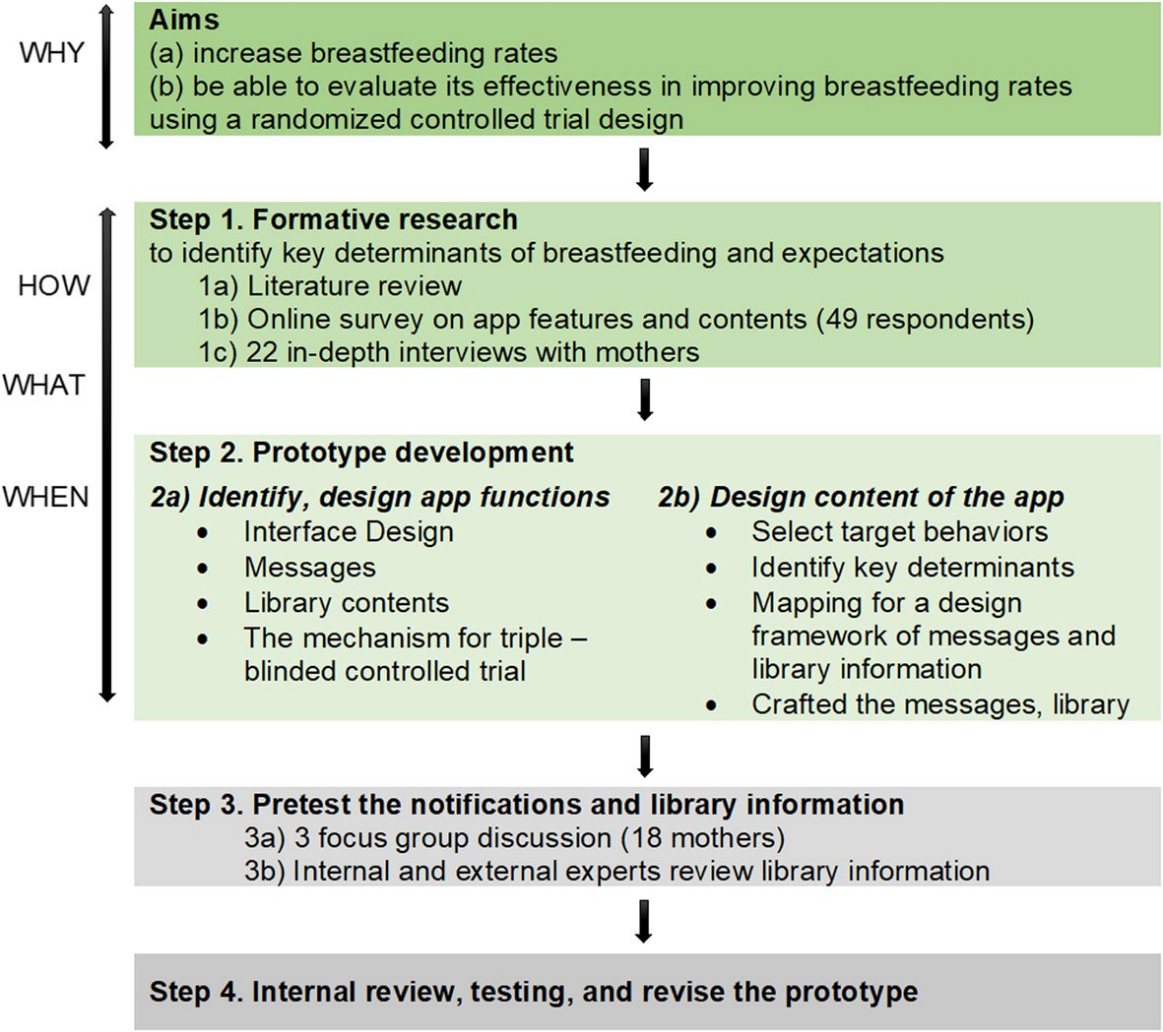
Process for developing BeBo mobile app, adapted from Mohr and Abroms [21, 24]

Combining both the behavioral intervention technology model for developing the app [21] and the steps for developing and pretesting a text messaging program for health behavior change [24], our process comprised the 4 steps shown in Fig. 1.

#### Step 1. Identify the determinants of breastfeeding and expectation on the app

##### Online survey on app features and contents

We sent an online survey link through a Facebook page to the contacts of a young researcher aged in her 30s, who was likely to have contact with the infant’s mothers. A total of 49 mothers and fathers responded to the survey. A questionnaire included information on the characteristics of respondents, time for installing the app, desires for app functions, information on maternal and child health care, and breastfeeding. The preferences for information on maternal and child health care were scored from 1 to 3, in which, 1 was the lowest and 3 was the highest. Data were collected using the Qualtrics system (Qualtrics, Provo, UT).

##### In-depth interviews with mothers

We conducted 22 in-depth interviews with mothers to explore the facilitators and barriers of breastfeeding. Mothers were asked to describe their feeding experience since they delivered the child. The interview’s guidelines were about what kind of food they feed to the child since birth, how they selected the feeding method, what made them select or change, their perception of the benefits of breastmilk and breastfeeding, challenges they meet during breastfeeding, and kind of supports they received during breastfeeding. The belief and practices on post-birth rituals to infant feeding which included solid food introduced were also asked.

Mothers who visited the two hospitals involved in the study were invited to participate in the interviews. To capture the widest range of perspectives possible, mothers were screened using a set of questions on the age of the child (less than one or more than 1 year old), experience with breastfeeding (exclusive breastfeeding or mixed with formula feeding), and mode of delivery (vaginal or cesarean while they waited for a health check-up in the hospital. Mothers were selected to ensure a variety of socio-economic groups and experiences and were invited for in-depth interviews after finishing their health check-ups. Interviews were undertaken either in the hospitals or the cafeteria nearby, the location chosen by the mothers. Each in-depth interview lasted 45–60 minutes. Selection processes and interviews were conducted by a researcher who had public health background and was experienced in qualitative study. After every four to five interviews, the researcher summarized the main themes and discussed with the team for selecting new mothers and revising questions’ guidelines until the information was saturated.

#### Step 2. Prototype development

We began the prototype development after completing the analysis of the results from the online survey and the 22 in-depth interviews. Regarding the app functions, we outlined desired functions which included pushed notifications, library content, and the mechanism for a triple–blinded controlled trial. We also created visual mock-ups with interface design reviewed by team members and revised. We selected target behaviors, identified key determinants of the behavior, and then mapped the push notifications to develop library content.

##### Messages or push notifications

We applied self-efficacy theories for developing behavior change messages because the intervention of the mobile app was more focused on individual approaches. Maternal breastfeeding self-efficacy (BSE) is reflective of a mother’s confidence in breastfeeding and is a modifiable factor [11]. The BSE theory postulates that women with higher BSE will have better breastfeeding outcomes [11]. These theories have been used widely in individualized interventions aimed at mothers. Breastfeeding self-efficacy items were a guide for developing the messages or notifications as they needed to be short, clear, and call for action. By tapping the notification, mothers opened the thread in the library information which determinants of BSE were explained in more detail.

##### Library information

Library information was developed based on the framework for messages and the Dennis BSE Scale [9]. Determinants of BSE are modifiable factors such as previous experience with breastfeeding, seeing others breastfeeding, learning about breastfeeding from others’ experiences, and asking for help from health professionals. Behavior change communication materials, and guidelines from the Vietnam Ministry of Health, the National Institute of Nutrition, the World Health Organization, and the Australian National Health and Medical Research Council Infant Feeding Guidelines were the main references for developing the content of the library information.

#### Step 3. Pre-test the designs, notifications, and library contents

##### Focus group discussions

After developing the prototype, we conducted three focus group discussions to elicit feedback regarding the designs, push notifications, and library contents and made modifications accordingly before usability testing.

The three focus group discussions with mothers at 24–36 weeks gestation (18 mothers) were held for testing the app designs and messages for the intervention group in both hospitals, both primiparas and multiparous mothers. We asked mothers to score each message from 1 (the lowest) to five (the highest) in terms of their preferences. The messages were categorized into four groups (1) Believing breastmilk is the best option for newborns until 6 months (benefits of breastmilk/colostrum to newborn, to mothers, (2) belief in having enough breastmilk for the baby; (3) involving husband, grandmothers, and healthcare providers support for exclusive breastfeeding, and (4) planning for breastfeeding exclusively.

User-friendliness of the messages, perceived relevance of messages, trust in messages, understanding of messages, and new information learned were guided for discussion after ranking [25]. They also provided an example of library content for making comments. Each focus group discussion was conducted for about 90 minutes.

Focus group discussions were undertaken in person by a Vietnamese team member who was working on maternal and child health with experience in both quantitative and qualitative research.

##### Experts review library contents

The library contents were internally reviewed by three Vietnamese researchers who were the team members. We also invited five external reviewers who were health promoters and counselors on nutrition and breastfeeding to comment on the library content. Two of them were from the two hospitals, one from an international non-government health organization, one from the Ministry of Health, and one from the Hanoi University of Public Health. All of them had at least 5 years of experience in their work.

#### Step 4. Prototype testing

Among people who had a smartphone in Vietnam, 41.1% use the iOS platform, and 57.5% use the Android platform. BeBo was developed to work on the iOS platform version 11 and Android platform version 7 or upper which covered 92.4% of iOS and 77.3% of Android users, respectively [19]. We tested the app among the team members with both platforms and intervention/control mode). We selected the eight most popular Android phone vendors in Vietnam for testing. Together with Apple, those vendors account for more than 95% of the mobile market share in Vietnam [19].

### Statistical analysis

#### Quantitative data

A descriptive analysis was conducted using SPSS software (IBM Corp. Released 2017. IBM SPSS Statistics for Windows, Version 25.0. Armonk, NY: IBM Corp). The percentage was used to summarize binary variables and mean ± standard deviation to summarize the score variables. All variables were internally consistent in scale directions, e.g., 1 = lowest, 3 or 5 = highest. A higher mean indicates a more accentuated level of concern or preference.

#### Qualitative data

All interviews and discussions were recorded and transcribed to text using Word. Two researchers coded the transcripts independently and sought a mutual agreement on the main themes used in the content analysis.

## Results

### Step 1. Determinants of breastfeeding and expectation on the app

#### Online survey on app features and contents

The majority of 49 respondents (7 fathers and 42 mothers) were from Hanoi (74%), where the study was conducted. They all had children under 5 years old.

##### Time to install the app

81% wanted to install the app during pregnancy, and 17% wanted to have it after delivery.

##### Desired app functions

Respondents preferred the app which helped to monitor child development, send notifications, and remind of upcoming events, had information on maternal and child care, and the ability to search for needed information. Posting questions, sharing information, discussing, or receiving notifications on those posts were less preferred. Some other functions were suggested such as liking the app with clinics/hospitals for pregnant care or child care or searching nearby pediatrics hospitals, receiving counseling from experts, music for mothers and newborns, etc.

##### Frequencies of receiving push notifications

Equal proportions (33%) of participants wanted to receive push notifications daily or 2–3 times per week and only less than a quarter of participants would prefer to receive push notifications once a week (22%).

##### Content of push notifications

Each notification should contain a piece of information, the more information the better (63%). Receiving repeated messages every 4 weeks was selected by nearly one-third of the participants (33%).

After discussion within the research group, we decided to design and install the app for mothers during a pregnancy instead of after delivery as originally planned. Push notifications were auto-generated three times per week during pregnancy and two times per week after delivery. Notifications were repeated every 4 weeks to make sure all mothers received the messages before delivery.

##### Information preferences

The preferred information was divided into three groups: maternal care, child care, and breastfeeding. For maternal care, participants were keen to receive information on mental health, birth preparation, nutrition, and living with their parents-in-law rather than monitor the gestational weight gain during pregnancy or have sex during pregnancy. For child care, participants liked the information on the management of child health during illness, immunization, and monitoring an infant’s progress rather than on feedings. During breastfeeding, mothers asked about nutrition for mothers, issues related to breastfeeding, and difficulties rather than expressing and storing breast milk or infant formula.

#### Qualitative research findings

Among 22 participants, three mothers were pregnant with their second child, 5 had a child aged under 6 months, 5 had a child aged 6–12 months, and 9 had a child over 12 months. About 7 of 22 mothers had exclusively breastfed, 12 had both breastfeeding with additional formula feeding and three had used infant formula only. Ten mothers had a vaginal delivery and the remainder had a cesarean delivery.

##### From mothers

Early initiation of breastfeeding was the most difficult for mothers, particularly among those who had a cesarean delivery. Someone else (grandparents, fathers, health care workers) took care of their newborn while mothers often found the milk did not “come in” in the first days.

“I had a cesarean section. There was no skin-to-skin contact. The doctors took the baby out of the operating room and bring to her grandmother. After the operation, I was alone in a postnatal room for the whole day. On the second day, I was in pain and could not move, I did not have any drops of milk, and I could not sit up. So, grandmothers took care of her and gave her only bottles”. (0304Text2).

When milk had “come in”, mothers had a lack of knowledge and experience in dealing with difficulties during breastfeeding and often turned to an easier option of formula feeding with support from family members.

“On day 10th, I had a fever, I didn’t know it was mastitis at that time. I could not express milk much. It was so painful. I clicked my tongue: “let’s feed her with formula, I don’t have much milk, she is hungry and crying. Pumping causes soreness in nipples, tiredness, shivering, and fever but not much milk is extracted”. So, I did not eat much, and ran out of milk after 20 days. I fed my baby with formula completely since then”. (0904Text4).

Some examples of difficulties during breastfeeding were their infant not focusing on breastfeeding, both playing and sucking at the same time, prolonged time taken for breastfeeding compared with bottle feeding; a child sleeping while breastfeeding, which did not happen with bottle feeds; a child ‘demanding’ feeds too often and the mother didn’t get enough sleep at night and breastfeed refusal. Mothers were also stressed and blamed themselves as “bad mom” when breastmilk was not yet ejected or not enough, newborns gained no weight, etc. Good preparation and planning for breastfeeding during pregnancy would help to adapt to breastfeeding.

“On the third day, there was still no milk as full as I thought. I began to panic and was afraid of not having enough breast milk. I remember when I attended an antenatal class, the counselor repeated again and again that “if you think you don’t have enough milk for the baby, you won’t have enough milk for real! You must be relaxed and comfortable!”. I called my sister for support and reassurance. I tried to reassure myself and not to feed with formula. In the evening of the third day after giving birth, the milk came in a lot”. (0904Text3).

##### From family members

The husband could help his wife in preparing food, cooking, and clean for the baby. However, most of them did not have a preference for either breastfeeding or infant formula and seemed to follow the mother’s or grandparent’s decision. Messages which focused on, or emphasized the love between the father–newborn and husband-wife would involve the husband in helping with breastfeeding.

“My husband said nothing, but he seemed to follow grandparents who supported stopping breastfeeding. He thought I would be tired from being woken -up many times to breastfeed the baby at night”. (0904Text1).

The mother-in-law is the most influential person. She was very helpful in caring for newborns and mothers as local birthing traditions did not support new moms doing housework in their first month after delivery. However, the differences in lifestyles and caring could create more stress for first-time mothers.

“After giving birth, I was very tired. My son was the first grandchild of a big family. His grandmother hugged and carried him almost all day. I was even not allowed to hold him. I could not have skin-to-skin contact for the first 12 hours as I wanted. When he cried, I told the grandmother to let me breastfeed him, however, she scolded me: “He′s hungry! How can you feed him when you have no milk!” (0904Text3).

Grandmothers often turned to use infant formula to enable them to feed a child whenever he/she cries, a preference for a “chubby” baby. They had also been exposed to infant formula advertisements on television. Involving family members in the preparation and planning for breastfeeding during pregnancy would support their breastfeeding decision and practice.

### Step2. The design, function, and content of the app

We used results from the literature review and the formative research above to identify and design the app functions.

#### 2a. App function and design

Being simple and easy to use and the ability to function without internet access were important criteria for the app functions.

The app function included (a) push notifications: a short message service (SMS) system to continue delivering information to mothers about target behaviors and encourage them to use the app; (b) library information which contains threads explaining the short messages. Two versions of the app were available, one for the intervention and the other for the control app. In the intervention version, the mother received messages and library information on breastfeeding and maternal and child health care. In the control one, no information on breastfeeding was given, the mother received messages and library contents on maternal and child health care only. In the trial, the researchers randomized the mothers into either of the version based on age, educational level, and parity [20].

The app Bebo allowed mothers to (1) Register; (2) Receive messages at a desired day and time in a week; (3) Reread the messages; (5) Touch the messages to open the related thread in the library content; (6) Read and search information in the library content. During the trial, the app needed to register with a password provided by the researcher for a randomized controlled trial study. This password was removed after the project ended.

##### Interface design

**Fig. 2:**
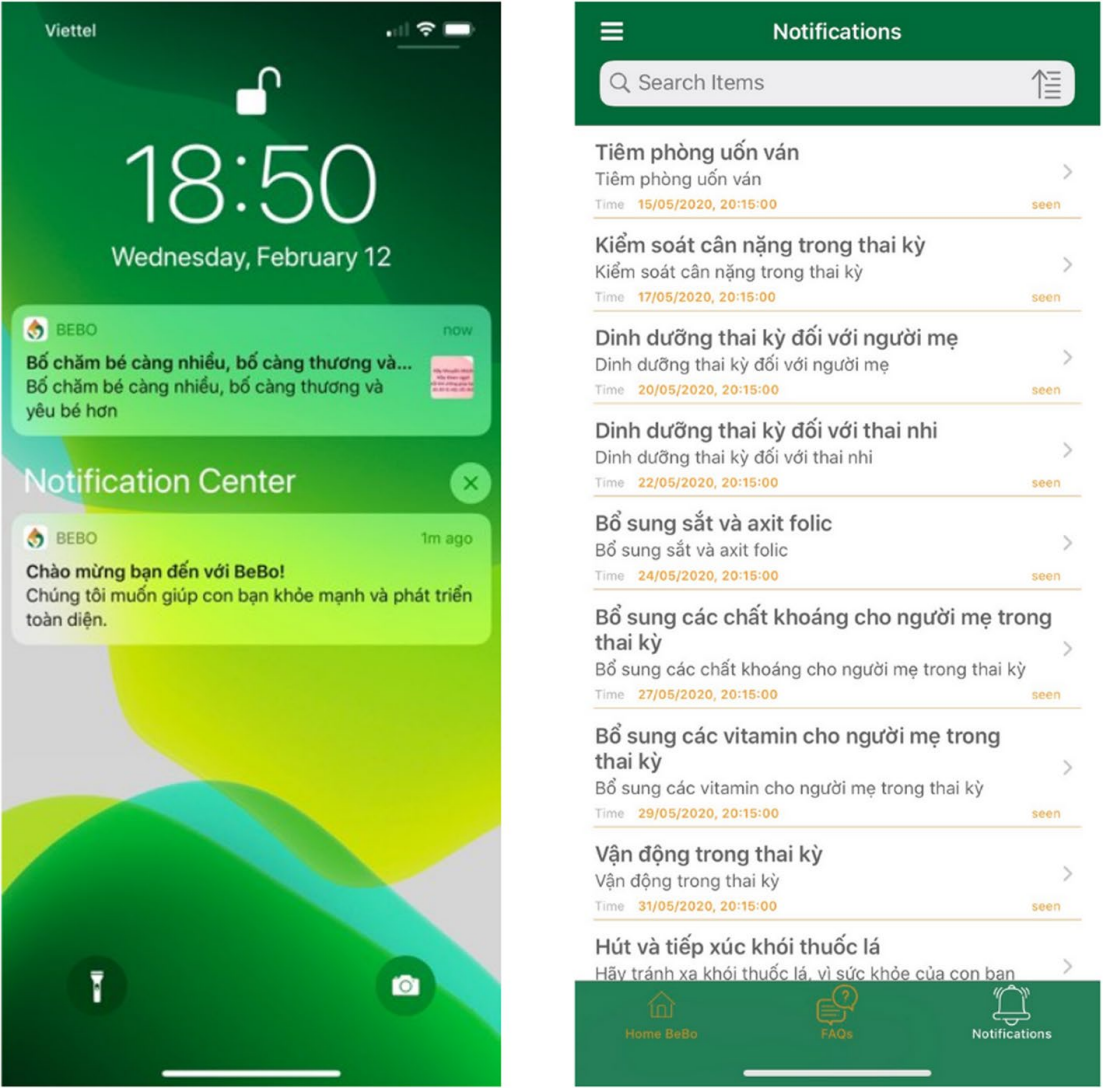
Home screen and notifications log

This theme refers to the design and layout, including consistency, location of icons, functions on each screen, font, color, density, placement, and images [26]. The graphic design was developed to be clear and intuitive to highlight the messages on breastfeeding. The logo of the Bebo app described a mother holding her baby and all were protected by a drop of breastmilk. Green was symbolic of nature and healthy growing up. Ochre could capture attention quickly, and evoke certain moods, it represented the stability of our environment and even influenced behavior and well-being (Fig. 2). A screenshot of log-in showed simple functions of library content, notifications, and search (Fig. 2).

##### Push notifications

The notifications conveyed messages on targeted behaviors. Users could tap the messages to open the related thread in the library information. Messages were designed to be re-readable at any time in the app [23], automatically generated, and sent to mothers’ phones without internet access three times per week during pregnancy and two times per week after delivery.

During pregnancy, the notifications were re-sent after every 4 weeks to make sure participants received a full set of 12 messages before delivery. After delivery, the messages were released according to key development milestones of the baby Users could tap messages on the home screen to access expanded content in the libraries.

Messages could be reread at any time in the app. They were about tetanus vaccination, weight control during pregnancy, maternal nutrition, folic acid supplementation, other micronutrient supplementation, physical activities, and smoking.

##### Library information

**Fig. 3:**
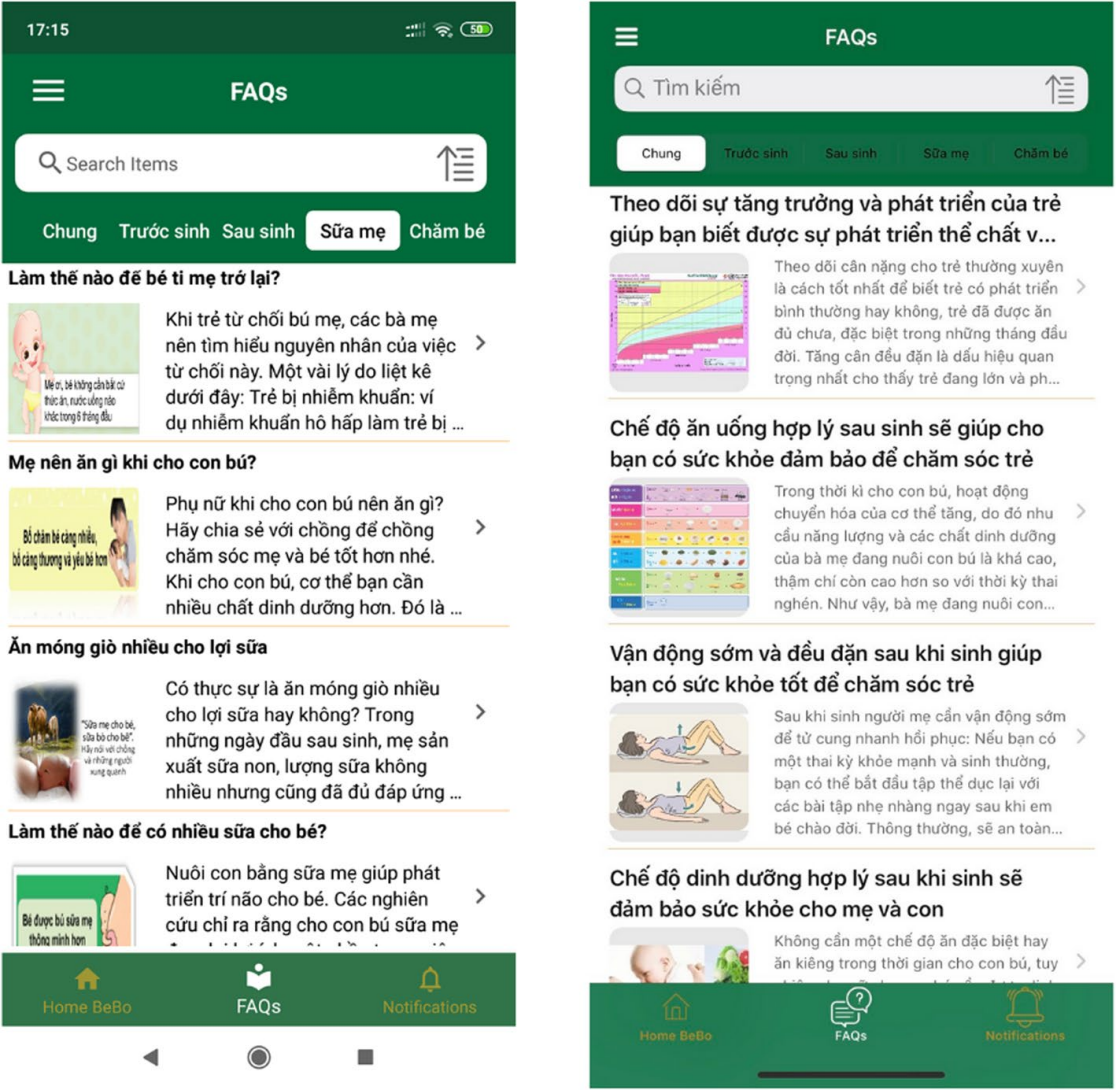
Library content of the intervention (left side) and control (right side) group

Each message was linked with one piece of information in the library (a thread). They were repeated every 4 weeks but the information in threads was different to provide more information and keep the mother reading. Each thread contained the message, the title of the thread related to the messages, a picture to illustrate messages or information in the thread, references, and links for further reading. The thread could be opened and read at any time without internet access. The library content for the control group also appeared in the intervention version. Library content for the intervention group did not appear in the control version (Fig. 3).

##### The design of the triple–blinded controlled trial

**Fig. 4:**
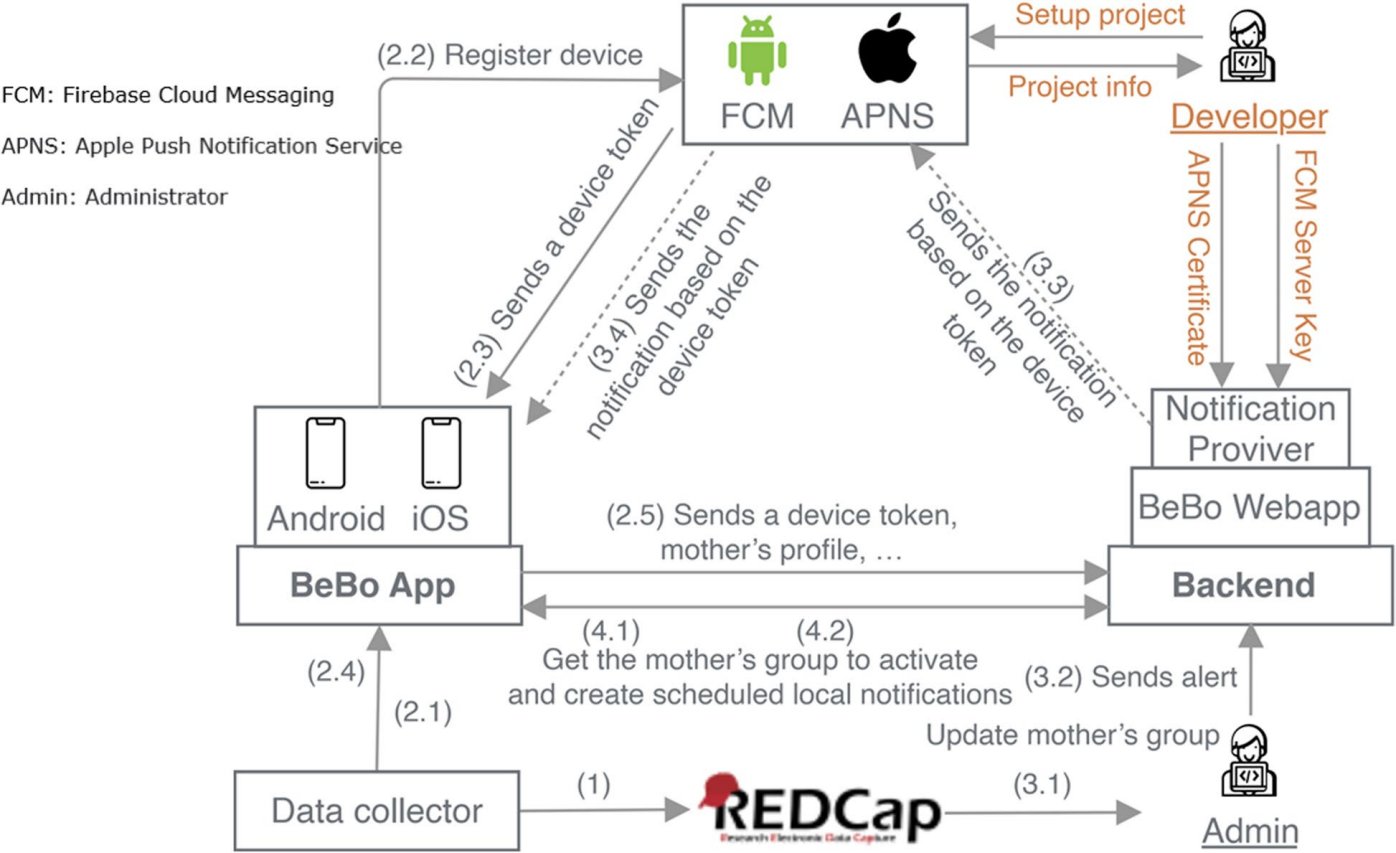
Architecture diagrams

The design of the triple–blinded controlled trial is shown in Fig. 4.

1. The data collector interviewed mothers using Research Electronic Data Capture (REDCap) and each mother was given a unique identifying number.
2. Registration
  2.1 The data collector team downloaded and installed the app and then launched the app for the first time to subscribe to push notifications with firebase cloud messaging or apple push notification service.
  2.2 The app registered the device to firebase cloud messaging or the apple push notification service.
  2.3 The firebase cloud messaging or apple push notification service accepted the app and sent it to the app as a device token.
  2.4 The data collection team updated the mother’s profile including the mother’s name, identifying number, phone number, and the desired date of the week’s notifications.
  2.5 The app sent the mother’s profile including the device token to the Webapp (the app was now waiting for activation of the version).
3. Randomization
  3.1 The administrator used information from REDCap (mother age, educational level, and parity) for randomizing the mother into either the control or intervention group. He then updated the mother’s group in their profile on Webapp to assign them either a control or intervention version.
  3.2 The administrator sent an alert to the mother to open the app for activation.
  3.3 The Webapp sent the notification to the mother app via firebase cloud messaging or apple push notification service based on the device token.
  3.4 The firebase cloud messaging or apple push notification service sent the notification to the mother app based on the device token.
4. Activation
  4.1 Mother touched or opened the notification to activate the app without notice of the app’s version.
  4.2 The app requested the mother’s group from Webapp to create the scheduled local notifications and finished the app registration and activation.

Only the administrator could extract information from REDCap and Webapp. In the follow-up phone call, the admin provided data collectors with name, identity number, phone number, and expected birthdate which were extracted from the Webapp. Data analysts would be provided with a data set without information on the control/intervention group.

#### 2b. Design content of the app

##### Messages or push notifications

We first identified target behaviors, then key determinants, and applied BSE theories for developing the messages and library content (Table 1). Barriers and facilitators to breastfeeding were identified from the review and the qualitative research above. The messages were targeted at not only mothers but also included fathers, mothers-in-law, or families [27].

**Table 1:** Example of the framework for developing messages

Program Objectives/Target behaviors	Key determinants (Theoretical domain)	Breastfeeding Self-efficacy of Mothers	Example of messages
**Before delivery**			
The mother intends to exclusively breastfeed until the introduction of solid food	Believing breastmilk is the best option for newborns until 6 months	Keep wanting to breastfeed	Breastfeeding is the best food for your babies in the first 6 months. The more you breastfeed, the more you bond with your newborn
Mother prepares for initiating early breastfeeding, skin-to-skin contact	Believing having enough milk, emphasizing early breastfeeding and cesarean section mothers	Determine that my baby is getting enough milk	All mothers, either cesarean or vaginal delivery have “early milk” to breastfeed their baby soon after birth
Mother involves family members (father, grandmothers) in exclusive breastfeeding	Involving husbands and grandmothers in preparing for exclusive breastfeeding	Comfortably breastfeed with family members present; tell when the baby is finished breastfeeding	With your husband’s support, the father-child will bond more.
	Planning for breastfeeding exclusively	Deal with the fact that breastfeeding can be time-consuming; Manage to keep up with the baby’s breastfeeding demands	Do you prepare for breastfeeding? Talk to your mother/mother-in-law about your plan for support
**After delivery**			
Mothers early breastfeed, early contact: place newborn infant on breast within 30 mins of birth	No prelacteal feed	Successfully cope with breastfeeding as I have with other challenging tasks	Congrats! The sooner, the more you breastfed your baby, the better
The mother continues exclusively to breastfeed until the introduction of solid food at 6 months	Having enough milk	Be satisfied with the breastfeeding experience	Be relaxed! Your body is going to produce enough milk
Mother delays the introduction of solid food	Able to breastfeed without pain/problems	Ensure that baby is properly latched on for the whole feeding; Manage the breastfeeding situation to satisfaction; Manage to breastfeed even if the baby is crying	If your baby is properly attached and suckling well, it will reduce nipple pain
The mother begins regular feeding of solid foods (anything other than breastmilk or formula) around 6 months of age	Commit to breastfeeding exclusive (practice)	Breastfeed without using a formula as a supplement; Finish feeding on one breast before switching to the other breast; Continue to breastfeed for every feeding	To keep your baby strong and healthy don’t give any infant formula feeds

##### Library information

Findings from the literature review and formative research were used as a guide to identify the content needed in the library. For example, we included information on managing a crying child [28, 29], addressing breastfeeding problems (e.g., cracked nipples, breast engorgement, mastitis), insufficient child suckling, or attachment, a lack of lactation, and postpartum fatigue [29–31]. Traditional foods and herbs which were believed to increase milk supplies from the in-depth interviews and did not contradict the guideline were included for cultural relevance.

Most of the messages and library content highlighted the ability to have early initiation of breastfeeding and exclusive breastfeeding among all mothers regardless of delivery mode.

### Step 3. Pre-test the notifications and library contents

The three focus group discussions with 24–36 weeks gestation mothers (18 mothers) were held for testing the app designs and the messages of the intervention group.

An example of scoring for each message and discussion is provided in Table 2. Overall, participants responded positively to the messages on the benefits of breastfeeding to newborn infants, particularly those on strengthening the immune system. The benefits of breastfeeding on increasing intelligence quotient /being smarter/to have better study were doubtful as an advertisement rather than based on evidence. Mothers were uncertain about the benefits of breastfeeding in preventing breast cancer and reducing anxiety and stress. However, uncertainty with the information would prompt them to open and read more in the library. Mothers suggested that “increasing mother-child bonding” was the most motivational factor for mothers to breastfeed their babies and should be included in the message.

**Table 2:** Example of testing messages scoring by mothers based on their preference (1 = lowest, 5 = highest)

	Messages	Mean	Std Deviation	Comments from respondents
A	**Benefits of breastmilk to a newborn**			
A1	Children who are exclusively breastfed have a stronger immune system, less illness	4.78	0.42	Similar to message A5
A2	Breastmilk is better than infant formular/cow milk	4.72	0.56	The second most-liked message^a^
A3	Breastmilk is the best food for your babies, healthy and strong, babies	4.67	0.67	Despite having a lower mean, this message was the most liked by respondents^a^
A4	Breastmilk has all nutrition and water your baby needs	4.61	0.59	Need to add “within 6 months”
A5	Children who are exclusively breastfed are stronger and have fewer hospitalization	4.50	0.96	Similar to messages A1
A6	Breastmilk strengthens the health of babies, reduces the risk of being overweight and having a chronic disease	4.39	0.89	“Not based evidence” – for promoting breastfeeding rather than based on evidence”
A7	Children who are exclusively breastfed in the first 6 months are smarter and taller	4.17	0.96	“Not immediate and direct benefits for babies. The benefit may be not true but it’s good because Vietnamese mothers are to expect their children to be smart and study well”
A8	Children who are exclusively breastfed in the first 6 months are more successful in their study	3.82	1.2	“not based evidence – for promoting breastfeeding rather than based on evidence”, “Not immediate and direct benefits for babies”. “It may not be true but It’s good because Vietnamese mothers expect their children to be smart and study well”
B	**Benefits of breastmilk to a mother**			
B1	Breastfeeding helps the mother to prevent breast cancer	4.5	0.83	3 votes as the most like but one think that it is not true^a^
B2	Breastfeeding helps the mother to reduce anxiety and stress	4.39	1.01	“Breastfeeding is much more difficult than infant formular. A lot of mothers are stressed because of lacking breast milk. So it’s not true” “Doubtful about this” “Doubtful about this”
B3	Breastfeeding helps the mother lose weight faster	4.28	1.1	3 votes as the most like but one thinks that it is not true^a^

^a^ Mother was asked which one of the above messages you like the most

To enhance the belief in having enough milk for the baby, mothers preferred messages which showed encouragement like “Do not panic if you do not produce enough milk on the first day. Colostrum is available since you are pregnant. Your baby needs only a small amount of milk each time” or “Relax and ensure that your baby is properly attached and suckling well, your body will produce more milk”. One mother said:

“I like this one most because many people don’t know this. I read a lot of documents before delivery and have experience with friends. However, the feeling of lacking milk and baby crying still push me to infant formula” (FGD2).

The messages had high-ranking, for example, “Do not discard colostrum, let your baby feed”, and “wash your hands with soap and water after changing a nappy and before handling your breasts”. “Start to breastfeed within the first hour or so of birth” was not at high ranking as it was affected by the mother-child separation from hospitals and they could do nothing.

Bonding between husband-wife and father-child was the most important motivated father for supporting the mother in breastfeeding and those messages were at high ranking than others. An example was “The more papa takes care of the baby; the more papa is fond of and loves the baby”. Most mothers thought that education on breastfeeding would be the best way for involving grandparents in support of breastfeeding. However, the messages involving grandmothers had a lower ranking compared with ones from other groups. The most liked message targeted at grandmothers was not directly related to breastfeeding: “Talk to your mother, mother-in-law about the food you want to eat after delivery”.

Messages on talking with health care providers were the lowest ranking among mothers. Most mothers were hesitant to talk with healthcare providers. A mother commented:

“Public hospitals will not support mothers. If you ask healthcare providers to do something, you would be scolded. Healthcare providers don’t like to be told what to do” (FGD1).

An example of library content was provided for mothers for discussion. They valued short and simple rather than evidence-based long and details content. The library information was internally reviewed by three Vietnamese researchers first and then by five external health educators on breastfeeding.

### Step 4. Internal review, prototype testing, and revision of the app

Modifications were made based on focus group feedback. Prototype testing to detect errors was conducted before the app went live. A total of eight iOS and five Android beta versions had been tested and revised. Some major errors were fixed including unable to install the app, unable to activate the app, search the algorithm, and update the mother’s profile. Some minor errors were fixed including larger font size, higher contrast color filters designed, mixed English and Vietnamese, spelling mistakes, and adjusting pictures in the app to fit with the screen. After prototype testing and adjustments, BeBo is being currently evaluated in a randomized clinical trial with the target population described.

## Discussion

We developed the BeBo App using both the behavioral intervention technology model for developing the app and the steps for developing and pretesting a text messaging program for health behavior change.

### App function

The app is relatively simple with text messages and library information. Another app developed for increasing breastfeeding among African Americans had almost the same technological components and had above-average usability [32]. As relevant and timely text messages were the most important element for engagement [22, 33, 34], we carefully followed steps for developing and pretesting text messages for health behavior change [24]. We received positive feedback on text messages and revised them carefully through group discussions. The frequency of sending messages, three-time during pregnancy and twice after delivery was based on mothers’ preference. Mothers could also select a day and time for receiving messages personally.

We understand that the interactive app feature is of interest and may increase engagement. However, we decided to not develop an app for monitoring the child’s growth, albeit it was one of the most preferred features reported by mothers. There are additional privacy considerations with receiving detailed information of this type. As we targeted to improve exclusive breastfeeding rates, the app was designed for mothers’ use until 6 months after birth. At this stage of development, attention to the increasing weight of an infant may drive a mother to formula feeding, whereas the public health emphasis is on overall development [25, 35].

Often mothers do not manage exclusive breastfeeding due to unsolved difficulties in breastfeeding. Therefore, emotional support along with providing appropriate knowledge is very important for lactating mothers. A function of mutual communication between a mother and lactation counselors built into the app is ideal. However, there is a lack of lactation counseling services in Vietnam so we cannot build this function.

### App content

A recent study found that the majority of the websites and apps on infant feeding were of poor quality and lacked evidence-based content [17, 36]. Information on the app is provided with credible and reliable resources from the Ministry of Health, the Australian National Health and Medical Research Council’s Infant Feeding Guidelines with the participation of experts on both nutrition and health promotion. We also involved mothers, the target group to transfer their needs and understand the information in the app. We hypothesized that well-developed apps can promote appropriate breastfeeding and support healthy growth and development.

### Messages to promote exclusive breastfeeding

Our findings on the determinants of breastfeeding were similar to previous studies in Vietnam. Mothers are aware of the need for breastfeeding [37, 38], and intended to practice [29, 39] and 96.4% tried to breastfeed their newborn [3]. However, early initiation of breastfeeding and exclusive breastfeeding within 6 months is still at low levels [3]. Our qualitative results suggested that knowledge was not sufficient for enhancing confidence in the ability of breastfeeding. There are opportunities for the app to include needed information. Besides the most preferred messages, we also included the messages which had low ranking as mothers were still “doubtful”. More detailed information to support and explain the messages was provided in library contents and mothers just tap the messages to open related information.

The desire to breastfeed and be a ‘good mother’ could create anxiety and stress for mothers who have found it difficult. A study in Vietnam reported that a mother who had higher breastfeeding self-efficacy was less likely to have postpartum depression [38]. A recent systematic review also showed that desire and failure to breastfeed or having experienced breastfeeding difficulties were detrimental to the mental health of mothers [40]. Likewise, our findings indicated that mothers cared much about the information on dealing with stress and preferred non-judgemental and supportive messages. We anticipate mothers who do not succeed in breastfeeding may stop using the intervention app which sends messages on breastfeeding only.

The support of partners and grandparents was important to mothers. A mother was more likely to continue exclusive breastfeeding at 4 and 6 months with a father’s support [41]. We encouraged mothers to involve influential people such as husbands, mothers/mothers-in-law, and healthcare providers. The importance and how to involve those peopled were provided in both messages and library content. Mothers were repeatedly reminded about asking for help from healthcare providers when they have any difficulties. The list of the hospitals with contact details and a further link to read/support were also provided in the library content.

However, those messages received low preferences compared with the other messages. Mothers were reluctant to ask healthcare providers for support on breastfeeding. Healthcare providers thought they were for treatment, and health information was delivered in a one-way style, with reliance on writing rather than counseling [42, 43]. On the one hand, the app which targeted mothers would be the most. On the other hand, other iterations to engage support persons should be considered in a future study.

The effectiveness of the app had been evaluated in a randomized controlled trial and resulted in the significance of improving early initiation of breastfeeding within 2 h and exclusive breastfeeding during the hospital stays among mothers who have a cesarean section [44].

### Limitations

There are several limitations to be considered with this project. The link to the online survey provided a sample of mothers who had a higher level of education and who lived in an urban environment. These respondents may not be typical of the general population. We used semi-structured questions to guide focus group discussions and in-depth interviews. However, divergent views may have been missed and we may receive possibly feedback from respondents than in the general population. Self-selection bias could have been possible given convenience sampling was used. Those who were interested in obtaining maternal and child care information and technological interventions may be more likely to participate in our study than the others. They may have a better knowledge of maternal and child health care as well as the ability in using smartphones. We considered all these aspects in developing the app.

## Conclusion

The ideal mHealth breastfeeding intervention is an app with text messaging and library content developed. The development followed a best practice approach, including the involvement of related stakeholders and grounding in behavior change theory. The mechanism to evaluate the effectiveness of mHealth interventions in rigorous, high-quality evidence by a triple–blinded controlled trial study was built. Further studies which use the mHealth approach may consider following those steps and experience for better design intervention to promote breastfeeding.

## Data Availability

The datasets used are available from the corresponding author upon reasonable request.
